# Iatrogenic right coronary artery dissection caused by diagnostic transradial cardiac catheterization

**DOI:** 10.1002/ccr3.1047

**Published:** 2017-06-19

**Authors:** David Baghdasaryan, Aram Nazaryan

**Affiliations:** ^1^ Department of Invasive and Interventional Cardiology Medical Center Nork Marash Yerevan Armenia

**Keywords:** Coronary spasm, diagnostic catheterization, iatrogenic coronary dissection, transradial approach

## Abstract

We are presenting rare, but life‐threatening complication of diagnostic coronary catheterization. To overcome the problem requires operator's experience and appropriate actions. We want to share our experience to interventionalists to be aware and ready to recognize and overcome such complication.

## Introduction

Percutaneous coronary intervention is an effective procedure of coronary revascularization, but it carries potential risk of iatrogenic complications in adjunction to expected benefit. It is important to foresee and recognize potential complications and be aware of their management. We present an illustrative case report of the RCA spasm and subsequent iatrogenic dissection, which was treated with prompt deployment of stents.

## Case Presentation

**Figure 1:**
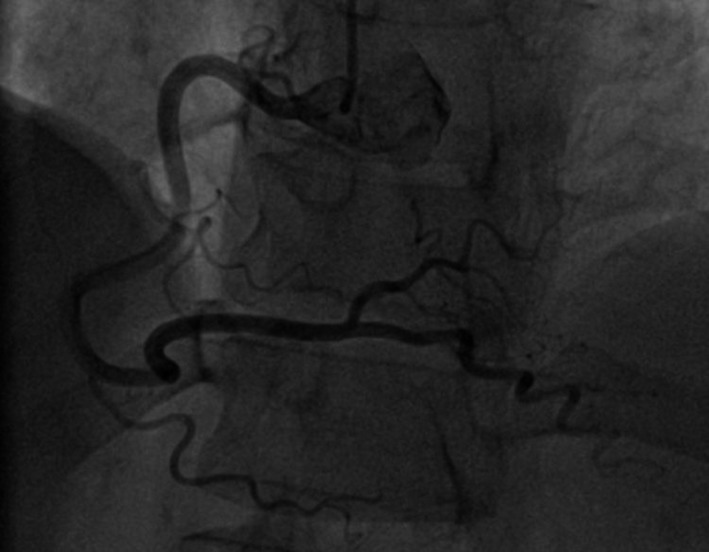
Normal right coronary artery.

**Figure 2:**
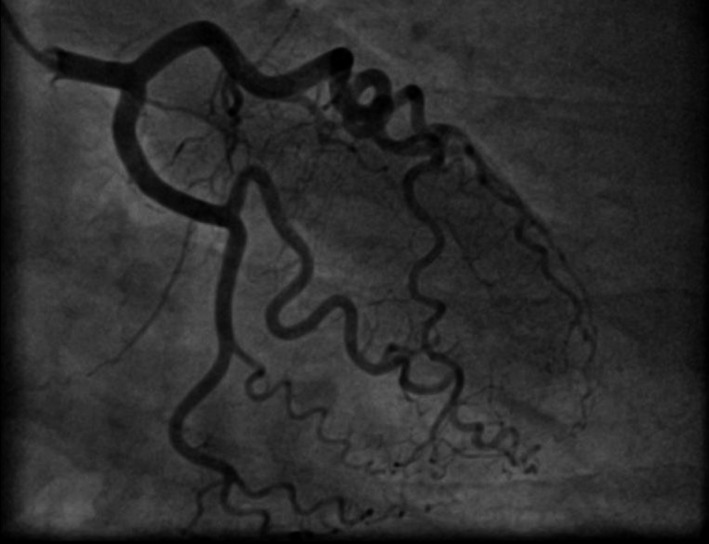
Normal left coronary artery.

**Figure 3:**
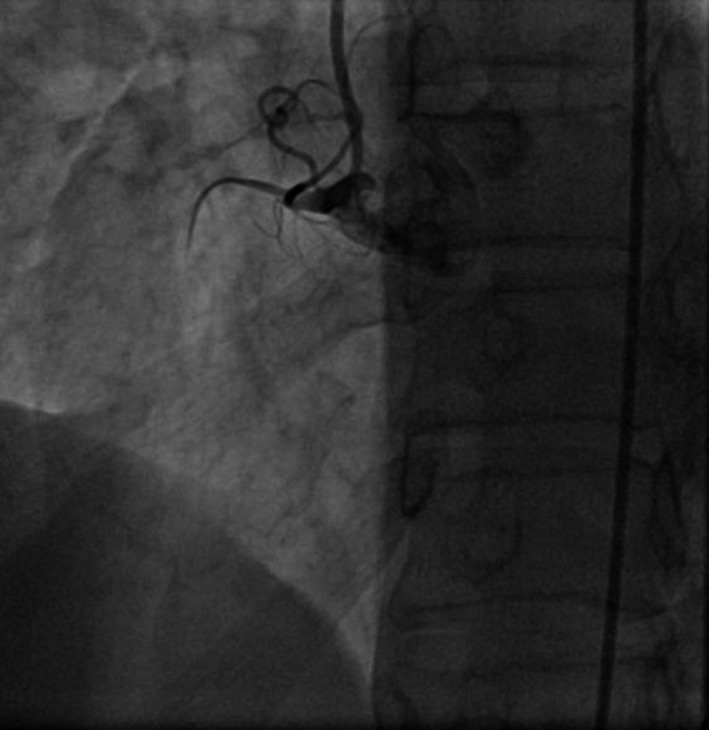
Occluded RCA in first injection.

**Figure 4:**
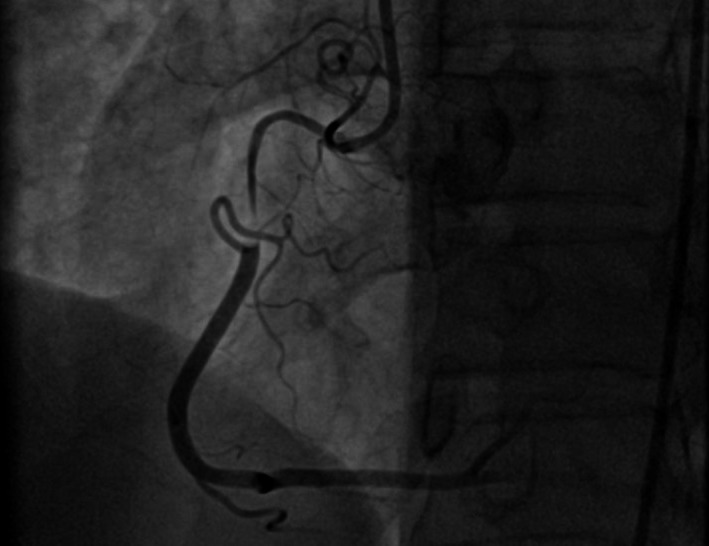
Visualized dissection after the second contrast injection.

**Figure 5:**
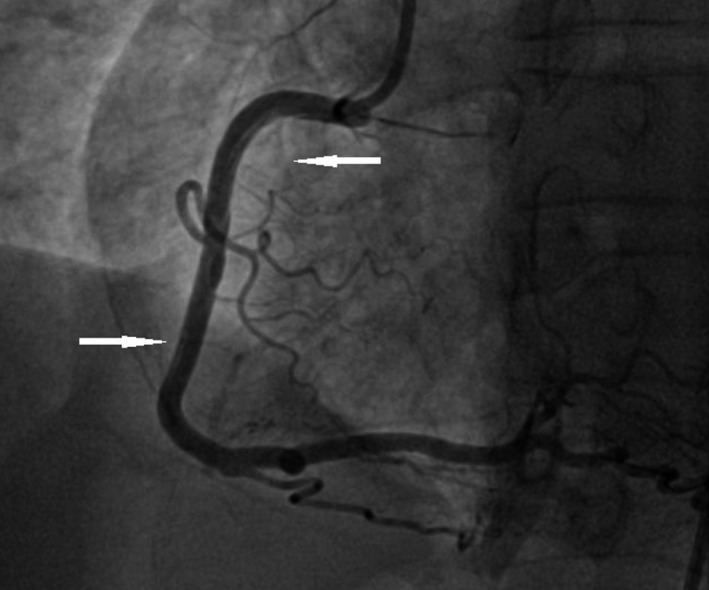
White arrows clearly show the dissection of the proximal and distal portions after guide wire insertion and balloon predilatation.

**Figure 6:**
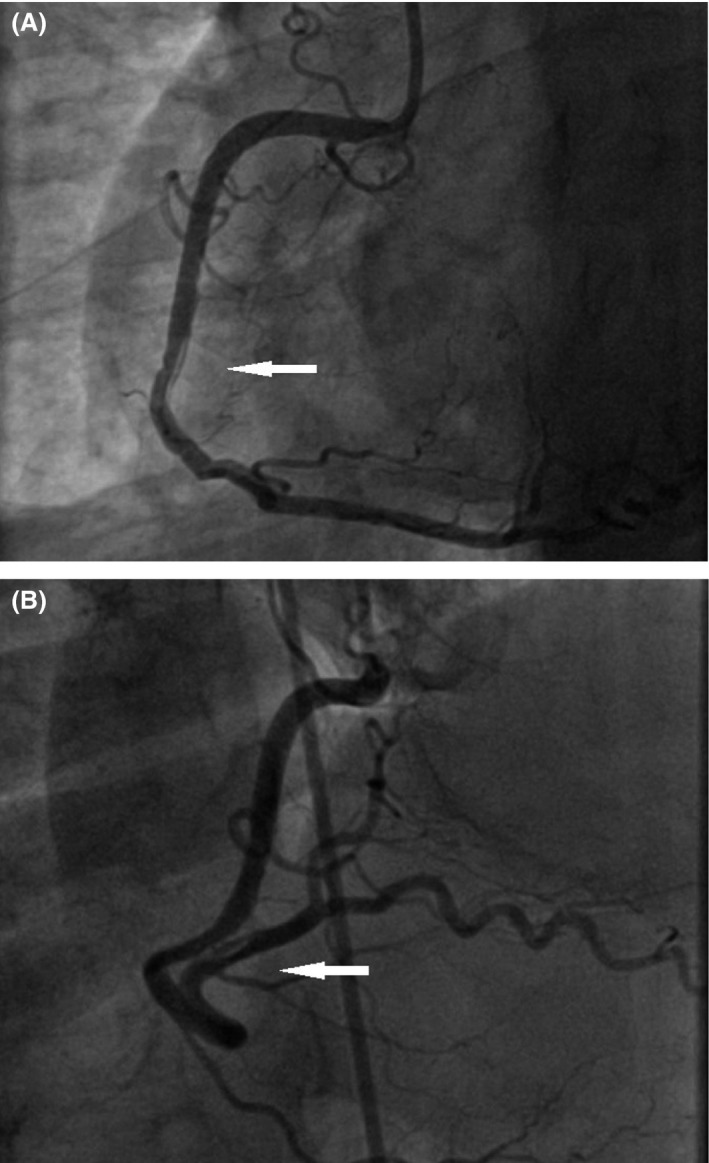
(A) White arrow shows the edge dissection after first drug‐eluting stents (DES) implantation. (B) White arrow shows the edge dissection after the second drug‐eluting stents (DES) implantation.

**Figure 7:**
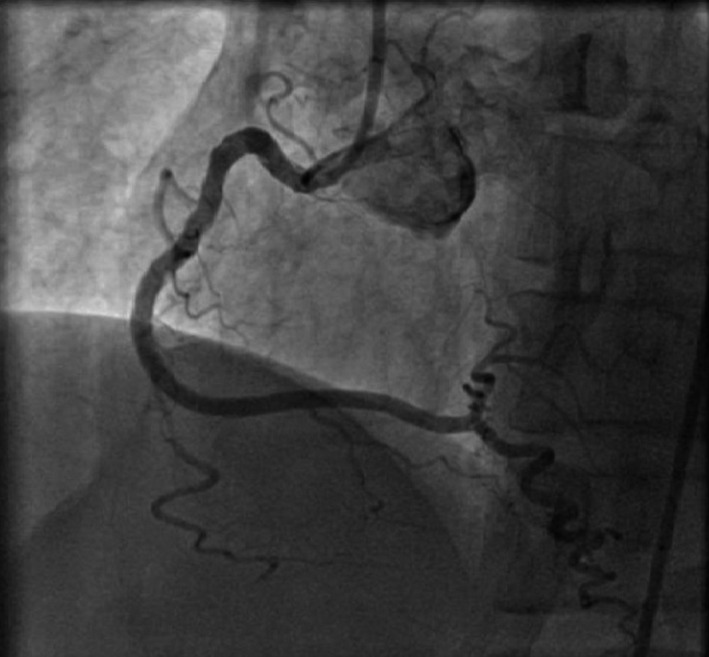
Final result after implanted three drug‐eluting stents (DES).

A 50‐year‐old woman was admitted to our hospital for diagnostic cardiac catheterization. She was initially referred to an outpatient clinic because of recent episodes of chest pain on exertion radiating to her left arm. Risk factors for coronary artery disease included uncontrolled hypertension and untreated hyperlipidemia with total cholesterol levels of 290 mg/dL and low‐density lipoprotein levels of 154 mg/dL. The patient's resting electrocardiogram (ECG) was normal and the clinical examination was unremarkable. Transthoracic echocardiography showed normal left ventricular function without wall motion abnormalities (ejection fraction 60%). The patient underwent a treadmill exercise stress test that was inconclusive for ischemia. Coronary angiography (CAG) was performed 2 days later via the right transradial approach. Following local anesthesia, the radial artery was punctured with a 20‐gauge needle and cannulated with a soft, 0.025″ straight guide wire. A 5 Fr, 23 cm radial sheath (Cordis Corporation, Miami, FL, USA) was placed. Intra‐arterial verapamil (2.5 mg), nitroglycerin (200 *μ*g) and 5000 units of unfractionated heparin were administered. Angiography of the RCA with a diagnostic 5 Fr right Judkins 4.0 catheter (Cordis) was normal (Fig. 1). The left coronary system was engaged with a diagnostic 5 Fr left Judkins 3.5 catheter (Cordis) and was also normal (Fig. 2). The patient transferred to the hospital room. An hour later growing chest pain suddenly started and blood pressure decreased from 110 mmHg to 80 mmHg. On the ECG ST elevation in leads II, III, and aVF. Patient was urgently transferred to the catheterization laboratory and repeated CAG was performed through the right femoral arterial access. Left anterior oblique projection showed acute occlusion of the proximal RCA (Fig. 3). Firstly was suspected a spasm of RCA, but after intracoronary injection of 500 *μ*g of nitroglycerin, blood flow partially restored to TIMI 1 with signs of coronary dissection (Fig. 4). Oxygen, fluids, and analgesia were administered. After careful interpretation of the angiographic images, the diagnosis of iatrogenic dissection of the RCA was made. The strategy was to conduct the guide wire in the RCA and seal the dissection via stenting. A 6 Fr JR 4 guiding catheter (Cordis, Warren, NJ, USA) was used and 0.014″ soft‐tip floppy Marker wire (BMW; Abbott Vascular, Santa Clara, CA, USA) was advanced through the RCA in the distal portion without any significant resistance. Predilatation was performed with a 2.25 × 10 mm semicompliant balloon (Ryujin™ Plus; TERUMO Corporation Terumo Medical, Tokyo, Japan) and TIMI 3 flow was restored (Fig. 5, white arrows indicate the site of dissection). Given the length of dissection, it was obvious that it should take more than one stent. Eventually, to completely cover the dissection from proximal to distal part ofthe RCA, it took three drug‐eluting stents implanted, XIENCE Xpedition 3.0 × 48 mm (Abbott Vascular), Biomime™ 2.75 × 37 mm (Meril Life Sciences Pvt. Ltd., Gujarat, India), and Orsiro 2.75 × 22 mm (BIOTRONIK AG, Bülach, Switzerland), respectively, (Fig. 6A and B, white arrow indicates the site of dissection). The final result was satisfactory (Fig. 7). The patient was transferred to the coronary care unit with hemodynamically stable condition. The next day cardiac enzymes were elevated and the patient was discharged from the hospital after few days.

## Discussion

Catheter‐induced dissection of a coronary artery is a rare but well‐recognized complication of CAG with a high mortality rate if it is left untreated 1, 2. The exact mechanism of iatrogenic coronary artery dissection is not clear. It results from mechanical injury to the arterial wall during catheter or wire manipulation, passage or deployment of an interventional device, forceful injection of contrast medium, balloon dilatation, or stenting 3. In our case, manual injection of contrast after intracoronary injection of nitroglycerin could be the cause of worsening the dissection. During stenting, additional aid had to intravascular ultrasound (IVUS), but in the absence of our clinic carrying conductor in the true lumen, unfortunately, was not been verified.

## Concluding Remarks

Several lessons can be learned from this case.

First, it obliges us to remember that CAG remains an invasive investigation with rare but life‐threatening complications. Indications for diagnostic angiography should therefore be sound, especially regarding the patients with stable angina. Many studies could be made (MRI scan or perfusion) for verification. Second, all catheters must be manipulated cautiously, especially when engaging the left main (LM). Injections should only be made when catheters are properly placed and when normal pressures have been identified. Third, in the unlucky situation of a iatrogenic LM dissection, prompt diagnosis, and therapy must be initiated, and the most experienced colleagues and surgeons should be alerted. Fourth, if available and if tolerated by the patient, IVUS can help confirm the correct position of the wire in the true lumen, determine the extension of the dissection, and guide stent sizing.

## Conflict of Interest

None declared.

## References

[1] Garcia‐Robles, J. A. , E. Garcia , M. Rico , E. Esteban , A. Perez de Prado , and J. L. Delcan . 1993 Emergency coronary stenting for acute occlusive dissection of the left main coronary artery. Cathet. Cardiovasc. Diagn. 30:227–229. 8269495 10.1002/ccd.1810300310

[2] Kennedy, J. W. , W. A. Baxley , I. L. Bunnel , G. G. Gensini , J. V. Messer , J. G. Mudd , et al. 1982 Mortality related to cardiac catheterization and angiography. Cathet. Cardiovasc. Diagn. 8:323–340. 7127459 10.1002/ccd.1810080402

[3] Tomassini, F. , A. Gagnor , and F. Varbella . 2011 Perforation of the sinus of Valsalva by guiding catheter during the percutaneous coronary intervention via the right transradial approach: a very unusual complication. Catheter. Cardiovasc. Interv. 78:888–891. 21523896 10.1002/ccd.23117

